# Polydopamine Nanoparticles-Based Three-Line Lateral Flow Immunoassay for COVID-19 Detection

**DOI:** 10.3390/bios13030352

**Published:** 2023-03-06

**Authors:** Zhe Liu, Chaoyu Cao, Haoyang Tong, Minli You

**Affiliations:** 1 Department of Rehabilitation Medicine, The First Affiliated Hospital of Xi’an Jiaotong University, Xi’an 710061, China; 2 The Key Laboratory of Biomedical Information Engineering of Ministry of Education, School of Life Science and Technology, Xi’an Jiaotong University, Xi’an 710049, China; 3 Bioinspired Engineering and Biomechanics Center (BEBC), Xi’an Jiaotong University, Xi’an 710049, China; 4 Department of Mechanical Engineering, Hongkong University, Hongkong 999077, China

**Keywords:** COVID-19, autonomous antibody, lateral flow immunoassay, artificial intelligence, polydopamine

## Abstract

Currently, the global trend of several hundred thousand new confirmed COVID-19 patients per day has not abated significantly. Serological antibody detection has become an important tool for the self-screening of people. While the most commonly used colorimetric lateral flow immunoassay (LFIA) methods for the detection of COVID-19 antibodies are limited by low sensitivity and a lack of quantification ability. This leads to poor accuracy in the screening of early COVID-19 patients. Therefore, it is necessary to develop an accurate and sensitive autonomous antibody detection technique that will effectively reduce the COVID-19 infection rate. Here, we developed a three-line LFIA immunoassay based on polydopamine (PDA) nanoparticles for COVID-19 IgG and IgM antibodies detection to determine the degree of infection. The PDA-based three-line LFIA has a detection limit of 1.51 and 2.34 ng/mL for IgM and IgG, respectively. This assay reveals a good linearity for both IgM and IgG antibodies detection and is also able to achieve quantitative detection by measuring the optical density of test lines. In comparison, the commercial AuNP-based LFIA showed worse quantification results than the developed PDA-based LFIA for low-concentration COVID-19 antibody samples, making it difficult to distinguish between negative and positive samples. Therefore, the developed PDA-based three-line LFIA platform has the accurate quantitative capability and high sensitivity, which could be a powerful tool for the large-scale self-screening of people.

## 1. Introduction

The global outbreak and rapid spread of coronavirus disease 2019 (COVID-19), caused by severe acute respiratory syndrome coronavirus 2 (SARS-CoV-2), has caused serious damage to the global economy and posed a serious threat to global public health security [1]. COVID-19 is highly infectious due to its main transmission methods of droplet transmission and contact transmission [2], and asymptomatic carriers of the virus increase its transmission range [3]. Early detection of patients with COVID-19 can stop the transmission chain of SARS-CoV-2 and control the current pandemic [4]. However, the concentration of antibodies or viral load is too low to be detected in the sample of patients with an early infectious stage, which reduces the sensitivity of the diagnostic methods [5]. Therefore, it is necessary to develop rapid, specific, and sensitive diagnostic methods for COVID-19 to effectively control the spread of the epidemic [6].

Currently, many detection techniques have been developed for COVID-19, among which nucleic acid amplification tests and immunoassays are the main tools for the clinical diagnosis of infections [7]. They have been both widely used in different settings of COVID-19 detection based on their properties. The polymerase chain reaction (PCR), as one kind of nucleic acid amplification test, has been the gold standard for COVID-19 diagnosis since it is highly accurate and specific [8]. However, it relies on large instruments and specialized personnel to perform the test [9]. In comparison, the immunoassay is simple and rapid [10]. For now, as the large-scale COVID-19 outbreak unfolds, the autonomous immunoassay method has attracted increasing attention, since it can avoid staff gathering and decrease healthcare expenditures [11]. Nevertheless, its insufficient accuracy limited it as an important tool for people to screen themselves for COVID-19 at home [12].

The ideal autonomous immunoassay should be inexpensive, rapid, easy to perform, and have high accuracy [13]. To obtain an early/sensitive diagnosis of COVID-19, many institutions have made efforts to develop effective methods for the rapid detection of SARS-CoV-2 antibodies and antigens [14]. SARS-CoV-2 antigen can be used for the early detection of COVID-19 patients [15]. Nonetheless, low viral loads are often observed in some COVID-19 patients, so antigen testing requires greater sensitivity [14]. IgM and IgG are produced by the immune system when the body is infected with COVID-19. IgG/IgM detection can provide accurate information on the severity of SARS-CoV-2 infection and the stage of illness [16]. IgM represents a patient who may be in the acute phase of infection, while IgG indicates a late infection or the presence of a previous infection [17]. Currently, the common methods used for immunoglobulins detection include enzyme-linked immunosorbent assay (ELISA), chemiluminescent immunoassay (CLIA), and lateral flow immunoassay (LFIA) [18]. Of these, ELISA with high sensitivity and specificity is the most frequently used strategy in hospitals and third-party detection institutions [19]. For example, an indirect ELISA uses purified S1 RBD and N protein as coating antigens to detect the duration and positivity of IgM, IgA, and IgG antibodies after infection [20]. However, ELISA not only requires strict experimental conditions and specialized personnel but also takes a long time, with an average detection time of 2~8 h [19]. In addition, a magnetic chemiluminescent enzyme immunoassay has been developed for the combined detection of IgM and IgG antibodies, which improves the detection performance of CLIA compared with that of single antibodies, but also increases the cost and requires a matching chemiluminescent instrument [21]. LFIA is a rapid diagnostic platform for paper-based detection and analysis in about 5 to 20 min, with the advantages of low sample volume, simple operation, and low cost [22]. Additionally, selenium nanoparticles have been used as labeled probes to prepare LFIA to detect IgM and IgG antibodies, which can be evaluated using the naked eye with 94.74% sensitivity and 96.23% specificity [23]. Another LFIA-based immunoassay uses selenium nanoparticles as a labeled probe. The test requires only 10 min to achieve the sensitivity and specificity of 93.33% and 97.34%, respectively [24]. However, many institutions still doubt the accuracy of their diagnostic results, due to their low sensitivity and poor accuracy [25]. A previous report has stated that the sensitivity of the rapid tests can be less than 20%, leading to large-scale underdiagnosis of COVID-19 [26]. Therefore, a colorimetric LFIA with high sensitivity is urgently needed for the accurate and large-scale screening of COVID-19.

To address the above issues, we developed a three-line LFIA immunoassay based on polydopamine (PDA) nanoparticles for the accurate and rapid detection of IgG and IgM. In addition, the developed colorimetric LFIA showed a higher sensitivity for antibody quantification in serum samples than the commercial AuNP-based LFIA. Therefore, we believe that the developed PDA-based three-line LFIA colorimetric platform with accurate quantification capability and high sensitivity can be a powerful tool for low-cost, rapid, and accurate immunoassays for the large-scale screening of COVID-19.

## 2. Materials and Methods

Dopamine hydrochloride, tetraethyl orthosilicate (TEOS), N-(3-dimethylaminopropyl)-N-ethylcarbodiimide hydrochloride (EDC), dimethyl sulfoxide (DMSO), bovine serum albumin (BSA), triton X-100, tween 20, and trizma base were purchased from Sigma-Aldrich (St. Louis, MO, USA). Sodium polystyrene sulfonate (PSS, Mw = 14,900) was purchased from Shanghai Zhenjun Biotechnology Co., Ltd. (Shanghai, China). Poly(allylamine hydrochloride) (PAH, Mw = 15,000) and polyvinylpyrrolidone (PVP, Mw = 10,000) were purchased from Shanghai Maclean Biochemical Technology Co., Ltd. (Shanghai, China). Ammonia solution (25%) was purchased from Tianjin Beilian Chemical Co., Ltd. (Tianjin, China). Sucrose was obtained from Tianjin Hedong District Chemical Reagent Co., Ltd. (Tianjin, China). Sodium chloride was purchased from Tianjin Tianli Chemical Reagent Co., Ltd. (Tianjin, China). Ethanol, methanol, and isopropanol were all provided by Tianjin Fuyu Fine Chemical Co., Ltd. (Tianjin, China). Sulfo-NHS was provided by Shanghai Yanchang Biochemical Technology Development Co., Ltd. (Shanghai, China). MES and HEPES were purchased from MP Biomedical. (Solon, OH, USA). Glycine was purchased from Biotech Bioengineering Co., Ltd. (Shanghai, China). SARS-CoV-2RBD antigen, mouse IgG, and goat anti-mouse IgG were all provided by Beijing Berseth Biotechnology Co., Ltd. (Beijing, China). COOH-PEG-Silane (MW_PEG_ 2000) was purchased from Yusi Co., Ltd. (Chongqing, China). 

### 2.1. Synthesis of Polydopamine Nanoparticles

10 mL of ethanol and 0.75 mL of ammonia were mixed with 22.5 mL of deionized water in a round bottom flask, and the solution was stirred in a water bath at 30 °C for 30 min. Dopamine hydrochloride solution (0.659 mmol dopamine hydrochloride dissolved in 2.5 mL deionized water) was then rapidly added to the solution and stirred for 24 h. The solution was mixed with acetone at a ratio of 1:2 and left to settle for 24 h. The supernatant was removed by centrifugation, and the precipitate was collected (8000 rpm, 10 min, 25 °C). The precipitate was washed three times with acetone and then redispersed in deionized water and stored at 4 °C.

### 2.2. Polyelectrolyte-Mediated Silica Coating

5 mL of polydopamine nanoparticles (PDA NPs) were added to 5 mL of a 2 g/L aqueous PSS solution with 6 mM NaCl and stirred vigorously for 3 h. The solution was centrifuged twice continuously (8000 rpm, 10 min, 25 °C) to remove excess magazines and resuspended in 5 mL of deionized water. Then, the PSS-modified PDA was added dropwise to 5 mL of a 2 g/L PAH aqueous solution containing 6 mM NaCl, stirred vigorously for 3 h, centrifuged twice continuously (8000 rpm, 10 min, 25 °C), and the solution was resuspended in 5 mL of deionized water. Next, 5 mL of PAH-modified PDA was added to 5 mL of a 4 g/L PVP solution and stirred overnight. The PVP-modified PDA was centrifuged (8000 rpm, 10 min, 25 °C) to obtain a precipitate that was dispersed in 0.2 μL of deionized water. After adding 1 mL of isopropanol and 0.46 mL of water with vigorous stirring, 1.43 mL of ammonia-isopropanol solution (3.84 vol%) was added to the system. 0.4 mL of TEOS-isopropanol (0.97 vol%) was added to the solution with gentle stirring, and the reaction was carried out for 2 h. PDA@SiO_2_ was centrifuged (8000 rpm, 10 min, 25 °C) three times and redispersed in methanol.

### 2.3. Surface Modification of Polydopamine Nanoparticles

COOH-PEG-silane was dissolved in methanol (50 μL, 10 mM) and then added to 950 μL of PDA@SiO_2_ with vigorous stirring for 2 h. After that, the solution was centrifuged at 8000 rpm for 10 min at 25 °C and then washed with methanol and deionized water twice. Finally, the PDA@PEG was dispersed with deionized water.

### 2.4. Antibody Conjugation to PDA NPs

200 μL of PDA@PEG solution was washed three times with 200 μL of DMSO-MES buffer (33% *v*/*v* DMSO, 20 mM MES) and resuspended in 150 μL of DMSO-MES buffer, and the solution was sonicated to a homogeneous photodispersion. 6 μL of 6 mg/mL EDC and 6 μL of 6 mg/mL NHS were added to the solution and sonicated continuously for 30 min. Next, 800 μL 20 mM MES buffer and 37.5 μL different concentration ratios of SARS-CoV-2 RBD antigen and mouse IgG solution (1:1, 1:2, 2:1, i.e., 18.75 μg:18.75 g, 12.5 μg: 25 μg, 25 μg:12.5 μg) were sequentially added to the system and incubated at 37 °C with 100 r/min in a shaker for 2 h. Finally, 30 μL of glycine stop buffer was added to the system and the resulting PDA-antigen complexes were washed with storage buffer (50 mM glycine, 0.1% NaN3, 0.03% *v*/*v* Triton), and the complexes were redispersed in storage buffer and stored at 4 °C.

### 2.5. Characterization of PDA NPs 

To characterize the morphology of PDA nanoparticles, we photographed them with a FEI Talos F200C transmission electron microscope (TEM) at an accelerating voltage of 200 kV. A Zetasizer Nano ZSE was used to determine the dynamic light scattering (DLS) and Zeta potential of PDA nanoparticles. A Nikon D90 camera was used to take images of PDA-based LFLA and AuNP-based LFIA. All measurements were performed under uniform room-temperature conditions.

### 2.6. PDA-Based LFLA Fabrication 

0.5 μL of 1 mg/mL SARS-CoV-2 anti-human IgG and 0.5 μL of 1 mg/mL SARS-CoV-2 anti-human IgM were immobilized on NC membranes for forming two test lines, and 0.5 μL 0.2 mg/mL goat anti-mouse IgG was immobilized on a NC membrane to form a control line. The prepared LFIA was placed in a drying oven to be dried at 37 °C for 2 h and then stored at 25 °C for further use.

### 2.7. Lateral Flow Immunoassay Detection

A series of standard solutions (0, 10, 50, 100, 500, and 1000 ng/mL) were prepared by stepwise dilution of IgG and IgM produced against the S protein and mixed with human serum. Next, 30 µL of the standard antibody solution was thoroughly mixed with the assay solution, which contained 10 µL of PDA probe solution and 60 µL of HSLF buffer (270 mm NaCl, 100 mm HEPES buffer, 0.5% w/v Tween20, 1% w/v BSA). The solutions to be tested were added to LFIA for IgG and IgM detection. For the AuNP-based commercial LFIA (Beijing Baisaisi Co., Ltd., Beijing, China), 30 µL of antibody solution was mixed by dilution with 60 µL of commercial buffer to perform the test. 15 min later, the results of the LFIA were captured by a Nikon D90 camera under the same conditions. Image J was used to measure the optical density values of the test line (T) and the control line (C), with the antibody concentration reflected by the grey value of the T line.

### 2.8. Software Development

The software for automatic quantification was developed using Python and the graphical user interface development framework PyQt5. The software contains two main parts. The first part is the image reading part, which is made up of an image read button and an image viewer. After pressing the image read button, users can read images from local storage and show the images on the image viewer. On the image viewer, users can choose the test line region. The second part is the quantification part. This part is composed of two select buttons for deciding the type of strip (IgG or IgM), a quantification button, and a result viewer. With the test line region and strip type selected, users can press the quantification button to show the concentration of the strip on the result viewer.

## 3. Results and Discussion

**Figure 1:**
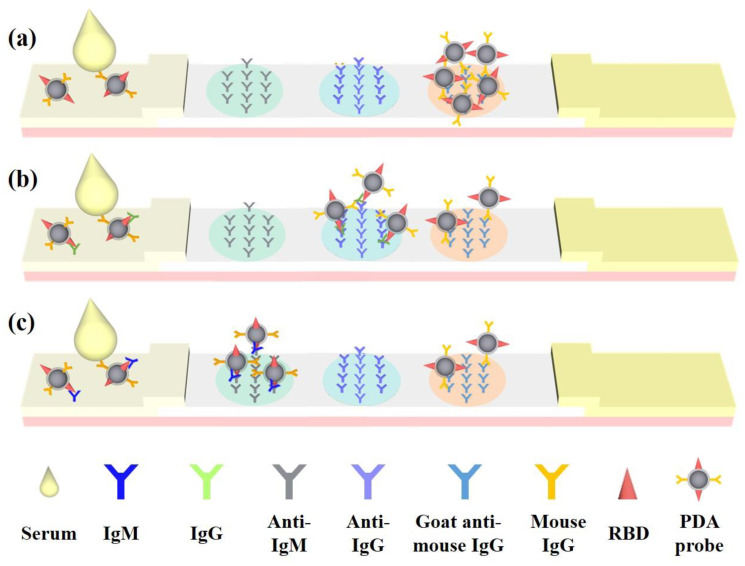
Detection principle of PDA-based three-line LFIA without targets (**a**) or with IgG (**b**) and IgM (**c**) in the serum, respectively.

Since the detection of IgM and IgG can provide information on the time course of viral infection, rapid detection of IgM and IgG can support the diagnosis and treatment of COVID-19 disease [27]. To achieve simultaneous detection of COVID-19 IgM and IgG, three test lines are designed for the PDA-based LFIA, including two test lines (anti-human SARS-CoV-2 IgM and anti-human SARS-CoV-2 IgG) and one control line (goat anti-mouse IgG) (Figure 1). Compared with the traditional colorimetric label (i.e., gold nanoparticles) used in most commercialized LFIA, the PDA nanoparticles enable a broader and stronger absorption band, resulting in improved detection sensitivity, which has been confirmed in our previous study [28]. To initiate the testing, 30 µL of serum is mixed with HSLF buffer and added to the sample pad to react with PDA probes. The resultant analyst-PDA complex can interact with the antibodies in the detection line, and the rest of the PDA probes can be captured by the goat anti-mouse IgG in the control line, driven by the capillary force of the absorbent pad. If the sample does not contain SARS-CoV-2 antibodies, the PDA probe does not accumulate at the test lines, and no significant color change can be observed, as shown in Figure 1a. If the sample contains IgG and IgM antibodies, these antibodies can first combine with the PDA probe co-labeled with RBD and mouse IgG before test lines. As the conjugated sample continues to flow, the IgM antibody-PDA complex can be captured by the IgM test line as in Figure 1b, and the IgG antibody-PDA complex can be captured by the IgG test line, as in Figure 1c, resulting in a color increase on the test lines. The remaining PDA probes continue to flow to the control line to be captured. This indicates that the PDA probes are sufficient, and this test is valid regardless of whether the sample is positive for SARS-CoV-2 infection.

**Figure 2:**
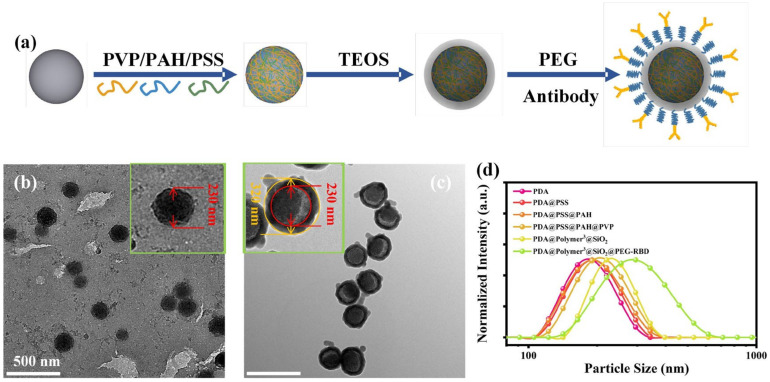
Synthesis and characterization of PDA probes. (**a**) Modification process of PDA@SiO_2_@PEG-Ab probes. TEM images of PDA (**b**) before and (**c**) after surface modifications. (**d**) Dynamic Light Scattering of PDA, PDA@Polymer^3^, PDA@Polymer^3^@SiO_2_, and PDA@polymer^3^@SiO_2_@PEG-Ab solutions.

Polydopamine is one kind of strongly adhesive polymer that is simple to prepare, environmentally friendly, and low cost [29]. Benefiting from these properties, PDA-based LFIAs are thus environmentally friendly and can be disposed of after use, and PDA NPs do not accumulate in the environment to result in a biosafety threat, such as AuNPs [30]. However, the adhesion property of PDA NPs makes them easily adhere to nitrocellulose membranes. Therefore, if antibodies are modified directly on the surface of PDA NPs, the resulting PDA probes will be mostly absorbed non-specifically in the test line, leading to false negative results. To solve this problem, we first formed three polyelectrolyte layers (i.e., PSS, PAH, and PVP) on the surface of the PDA NPs using a layer-by-layer assembly technique. Then, TEOS was added to form a dense silica layer to reduce the adhesion of PDA NPs. Following, COOH-PEG-Silane was modified on the surface of the PDA NPs to further reduce the adhesion of the PDA NPs and provide carboxyl groups for subsequent antibody modifications. Finally, the antibodies were incubated with PDA NPs to obtain PDA probes, as shown in Figure 2a. We performed TEM and DLS to characterize the size change of PDA nanoparticles during the modification process to verify whether PDA probes were successfully modified. From the TEM images, it was observed that the particle size of PDA NP before and after surface modification increased from 230 nm to 320 nm, and the significant increase in nanoparticle size indicated the successful preparation of the core-shell structure as shown in Figure 2b,c. Meanwhile, the DLS results verified that the size of PDA NPs kept increasing after successive surface coatings, which also confirmed the successful surface modification of PDA NPs as shown in Figure 2d.

**Figure 3:**
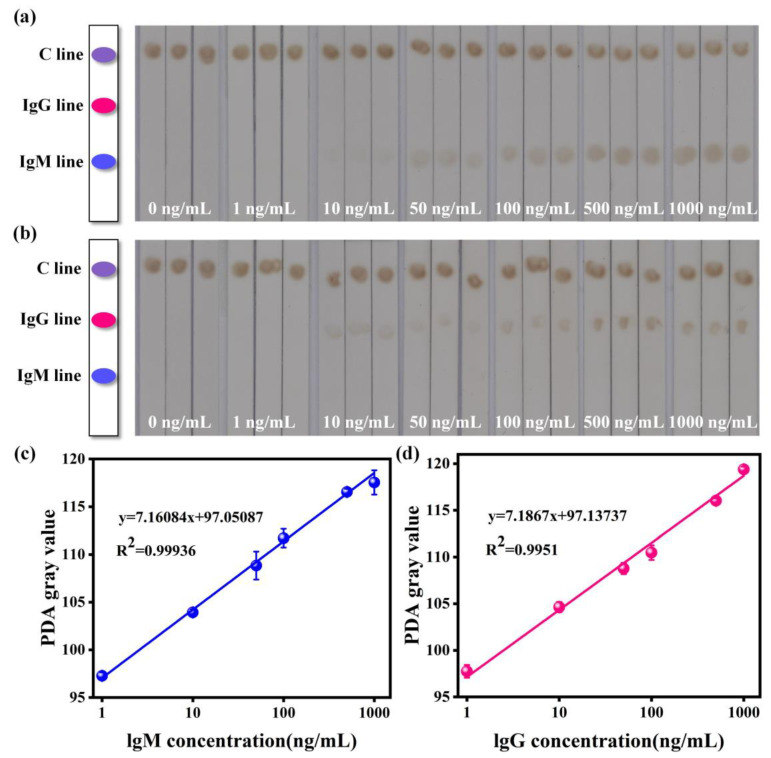
Standard curves of PDA-based three-line LFIA for COVID-19 IgG and IgM. (**a**) The images of the PDA-based three-line LFIA for COVID-19 IgG at different concentrations. (**b**) The images of the PDA-based three-line LFIA for COVID-19 IgM at different concentrations. (**c**) The standard curve of the PDA-based three-line LFIA for COVID-19 IgG at different concentrations. (**d**) The standard curve of the PDA-based three-line LFIA for COVID-19 IgM at different concentrations.

To evaluate the sensitivity of IgG and IgM antibodies detection by PDA-based three-line LFIA, we spiked serum samples with different concentrations of COVID-19 IgM and IgG antibodies. Here, we simulated the samples with different antibody concentrations of COVD-19 patients by mixing IgM and IgG in serum, respectively, while setting a concentration gradient for IgM or IgG antibodies for detection (0, 1, 10, 50, 100, 500, and 1000 ng/mL). The detection results were taken under the same environmental conditions to be read out. As shown in Figure 3a,b, the color of the test lines on the three-line LFIA gradually deepened as the concentration of IgG and IgM increased, wherein the IgG and IgM can be specifically identified by the corresponding test lines. To quantify the detection results accurately, we used the Image J software to measure the optical density of test lines and obtain the standard curves for IgG and IgM detection. The standard curve showed that the limits of detection of IgM and IgG by three-line LFIA were 1.51 ng/mL and 2.34 ng/mL, respectively. The linear correlations were obtained as IgM: *y* = 7.16084*x* + 97.0.5087 (R^2^ = 0.99936) and IgG: *y* = 7.1867*x* + 97.13737 (R^2^ = 0.9951), as shown in Figure 3c,d. It is proven that this method has a good linear relationship in the detection of COVID-19 antibodies and can be used for quantitative detection. It is worth noting that the PDA-based three-line LFIA developed here exhibits higher sensitivity compared to other colorimetric LFIAs (Table 1), which can be used for not only visual qualitative judgments but also quantitative detection with commercial optical densitometry to adapt for large-scale outbreak screening. 

**Figure 4:**
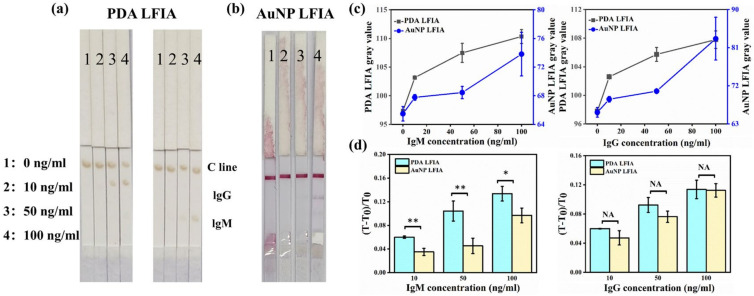
Comparison of PDA-based LFIA and AuNP-based LFIA for COVID-19 IgG and IgM detection. (**a**) Detection results of PDA-based three-line LFIA for IgG and IgM with different concentrations. (**b**) Detection results of AuNP-based LFIA for IgG and IgM with different concentrations. (**c**,**d**) Comparison of the quantification results of COVID-19 IgM and IgG detection. *p* < 0.05 indicates significant difference (*) and *p* < 0.01 indicates extremely significant difference (**).

To evaluate the accuracy of PDA-based three-line LFIA detection better, we compared the detection performance of PDA-based LFIA and the most widely used commercial AuNP-based LFIA for SARS-CoV-2 IgM and IgG antibodies. Here, we detected and quantified the results of samples containing different concentrations of SARS-CoV-2 IgM and IgG (0, 10, 50, 100 ng/mL) using the two methods mentioned above. In comparison with the PDA-based LFIA, the AuNP-based commercial LFIA could barely detect low concentrations of COVID-19 antibodies, as shown in Figure 4a,b. The quantitative results of AuNP-based commercial LFIA displayed a weak linear relationship, as shown in Figure 4c,d, especially for SARS-CoV-2 IgM samples. Here, T_0_ represents the gray value of T lines in the negative group (i.e., the 0 ng/mL group), and T represents the gray value of T lines in the positive group (i.e., the 10, 50, and 100 ng/mL groups). Therefore, the PDA-based LFIA was proven to have a better quantitative capability for serum samples compared with the AuNP-based commercial LFIA. Importantly, both methods require only 15 min for the detection process. Thus, the PDA-based three-line LFIA is more advantageous in terms of accuracy. However, the detection of IgG and IgM-based antibodies requires the collection of a serum sample, which can be uncomfortable for users and may lower the test’s overall acceptability.

**Figure 5:**
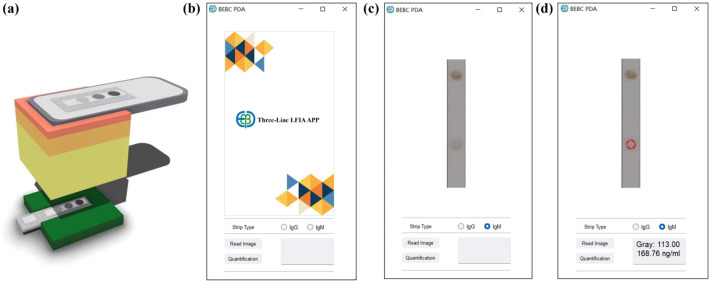
Software design for quantification of the three-line LFIA. (**a**) A Lego block-assembled smartphone reader for controlling illumination. (**b**) Main page of the designed software. (**c**) Select a page, including importing a photo and choosing the detected antibody type. (**d**) Result page shows the test line area extraction result (red dashed-line circle) and quantification result in the right corner.

To make it convenient for users to easily perform the quantification, we developed software with the functions of test line extraction and concentration calculation. After taking a photo of the three-line strip with the reader (Figure 5a), users can get quantification results from the software after sending the picture to the computer. The main page of the software contains three function buttons, “Strip Type,” “Read Image,” and “Quantification,” as shown in Figure 5b. “Strip Type” allows quantification of both IgG and IgM combined antibodies, and the “Read Image” button is used to import images from external storage, as shown in Figure 5c. We designed two manual steps to choose the test line area. The first step is to click the mouse on the position of the test line center, and the second step is to drag the mouse to generate a circle enclosing the test line. With the chosen test line, the “Quantification” button can show the mean gray value and detected concentration according to the above IgG or IgM linear correlations, as shown in Figure 5d. The software can be deployed both on cloud servers for remote virtual healthcare and on a personal computer for self-diagnosis. 

## 4. Conclusions

In conclusion, we successfully developed a PDA-based lateral flow immunoassay technique for the accurate and large-scale screening of COVID-19. Compare to other LFIA methods for COVID-19 diagnosis [33], two innovative points were presented in this study. One is that the brown PDA with a broader and stronger visible absorption band presents higher sensitivity compared to AuNP. The other is that the developed PDA three-line LFIA successfully achieved the detection of IgG-IgM combined antibodies, providing the possibility of accurate and large-scale screening of COVID-19. Therefore, we envision that the PDA-based, three-line LFIA could be a powerful tool for outbreak control and self-detection.

## Figures and Tables

**Table 1:** Results comparison of colorimetric LFIAs for IgG and IgM detection.

Targets	Probe	Limit of Detection (ng/mL)	Linear Range (ng/mL)	Test Time (Minutes)	References
IgG	AuNP	10^2^	200~10^4^	15	[31]
IgM/IgG	Selenium NP	60/20	60~10^3^/20~10^3^	10	[23]
IgM/IgG	Selenium NP	20/5	20~10^2^/5~10^2^	10	[24]
IgM/IgG	ePAD	10^3^	10^3^~5 × 10^4^/10^3^~5 × 10^4^	-	[32]
IgM/IgG	PDA	1.51/2.34	1~10^3^/1~10^3^	15	this work

## Data Availability

The data presented in this study are available on request from the corresponding author.
